# Bilateral Functional Connectivity of the Basal Ganglia in Patients with Parkinson’s Disease and Its Modulation by Dopaminergic Treatment

**DOI:** 10.1371/journal.pone.0082762

**Published:** 2013-12-20

**Authors:** Simon Little, Huiling Tan, Anam Anzak, Alek Pogosyan, Andrea Kühn, Peter Brown

**Affiliations:** 1 Nuffield Department of Clinical Neurosciences, John Radcliffe Hospital, University of Oxford, Oxford, United Kingdom; 2 Sobell Department of Motor Neuroscience & Movement Disorders, UCL Institute of Neurology, London, United Kingdom; 3 Department of Neurology, Charité, University Medicine Berlin, Berlin, Germany; University of Toronto, Canada

## Abstract

Parkinson’s disease is characterised by excessive subcortical beta oscillations. However, little is known about the functional connectivity of the two basal ganglia across hemispheres and specifically the role beta plays in this. We recorded local field potentials from the subthalamic nucleus bilaterally in 23 subjects with Parkinson’s disease at rest, on and off medication. We found suppression of low beta power in response to levodopa (t_22_ = −4.4, p<0.001). There was significant coherence between the two sides in the beta range in 19 of the subjects. Coherence was selectively attenuated in the low beta range following levodopa (t_22_ = −2.7; p = 0.01). We also separately analysed amplitude co-modulation and phase synchronisation in the beta band and found significant amplitude co-modulation and phase locking values in 17 and 16 subjects respectively, off medication. There was a dissociable effect of levodopa on these measures, with a significant suppression only in low beta phase locking value (t_22_ = −2.8, p = 0.01) and not amplitude co-modulation. The absolute mean values of amplitude co-modulation (0.40±0.03) and phase synchronisation (0.29±0.02) off medication were, however, relatively low, suggesting that the two basal ganglia networks may have to be approached separately with independent sensing and stimulation during adaptive deep brain stimulation. In addition, our findings highlight the functional distinction between the lower and upper beta frequency ranges and between amplitude co-modulation and phase synchronization across subthalamic nuclei.

## Introduction

The basal ganglia are comprised of a distributed network of subcortical nuclei with well described anatomical inter-connections and connections to and from the cerebral cortex, thalamus and brainstem [1]. These anatomical links are complemented by a flexible functional connectivity characterized in part by oscillatory synchronization [2], [3]. Elucidating the behavior and structure of such oscillatory activity is important for understanding basal ganglia function and for identifying reliable biomarkers of disease states that could be used to inform treatment.

Recordings from deep brain stimulation (DBS) electrodes in patients with Parkinson’s disease (PD) demonstrate excessive beta oscillations (13–32 Hz) throughout the basal ganglia and have shown that these beta oscillations are suppressed by movement, levodopa and by DBS [4]–[8]. Increases in beta power, recorded from a single site, are indicative of increased spatial-temporal neuronal synchrony in the localised vicinity of the recording electrode. Many reports have highlighted the correlation between local beta synchronization (and its surrogates), and clinical severity across sides, both with respect to the off-levodopa state and to levodopa-induced changes in both synchronization and clinical impairment [9]. It has also been found that there is bilateral coherence in the beta range between subthalamic nuclei (STNs) in the majority of PD patients when studied off medication [10]. This demonstrates that synchrony within the beta band is not only present at the local mesoscopic level but also occurs at the macroscopic level, resulting in a widespread distributed beta network. Inter-hemispheric coherence has correspondingly been found at the cortical level in PD and been shown to be suppressed by levodopa administration [11]. Whether, the bilateral subcortical beta network is similarly modulated by dopamine is as yet unknown and investigated in this study.

Previous investigations have often considered the beta band as a single functional unit, however there is mounting evidence of dissociable functional characteristics between low beta (13–20 Hz; beta 1) and high beta (21–32 Hz; beta 2) activities, with regard to movement induced desynchronisation [12] and suppression by levodopa [13]–[15]. Studies considering the different beta subbands have thus far only examined local synchrony within a hemisphere and it is therefore unknown whether there is a differential effect of dopamine on interhemispheric subcortical coupling in the low and high beta subbands. This too is investigated in the current study.

Finally, coherence, although a sensitive and well validated method for quantifying functional connectivity between different sites, is partly limited by being a composite measure which conflates both phase and amplitude correlation. A further pertinent question therefore, which we address here, is whether coherence can be deconstructed into independent phase synchronization and amplitude co-modulation, and whether these are similarly affected by dopamine? Addressing this question may help clarify the fundamental mechanisms employed by the brain for long distance connectivity at subcortical levels, and may also have practical implications. Recent work has shown the potential benefits of using beta oscillations to control the delivery of DBS [16], [17]. In PD patients this was achieved with unilateral DBS in which stimulation was delivered in response to beta amplitude threshold crossing. Advancing aDBS towards clinical application however will require a better understanding of how the basal ganglia systems on the two sides interact. Specifically, it remains to be determined how best to trigger bilateral adaptive stimulation and whether this would be optimally achieved by synchronous or asynchronous (independent) stimulation across the two sides. The identification of strong bilateral amplitude co-modulation of subcortical beta activity might encourage trials that involve a simpler, unilateral sensing of beta amplitude that then controls bilaterally synchronous DBS.

In this study we investigate a cohort of PD DBS patients on and off medication and analyse STN LFPs in an attempt to characterize the bilateral subcortical functional connectivity of the basal ganglia in the two beta sub-bands and their response to dopamine. In addition, we aim to deconstruct bilateral coherence into independent amplitude co-modulation and phase synchronization to ascertain which is driving the subcortical beta network and whether these aspects of connectivity have a differential response to dopamine.

## Materials and Methods

### Patients and Surgery

We investigated the bilateral connectivity between STNs in a cohort of 23 subjects with PD on and off levodopa. This comprised 10 subjects who were studied in the United Kingdom (UK) and 13 patients who were recorded in Germany. All subjects had advanced idiopathic PD with motor fluctuations and/or dyskinesias. The average age was 59±1.9 years and the preoperative score on the motor section of the Unified Parkinson’s Disease Rating Scale (UPDRS), [18] was 42±3.1 off medication and 17.3±2.4 on medication. The mean disease duration at the time of surgery was 11.5±0.7 years. The mean equivalent levodopa dosage was 1220±132 mg at the time of surgery. UK patients underwent bilateral DBS surgery on the STN as previously described [19]. The German operative procedure was similar [7]. Eighteen of the subjects have been previously reported as part of earlier studies; nine in [7], two in [20], one in [21] and six in [22].

### Ethics Statement

All subjects gave their informed written consent to take part in the study, which was approved by the National Hospital for Neurology & Neurosurgery and Institute of Neurology Joint Research Ethics Committee, London UK and the ethics Committee of the Charité, University Medicine Berlin.

### LFP Recordings

All subjects were recorded after overnight withdrawal of antiparkinsonian medication (off state) and following levodopa challenge equivalent to their standard morning medication (on state, minimum dose 100 mg) in the post-operative period (days 3–7), prior to battery and stimulator implantation. Improvement with medication was confirmed through assessment of finger tapping, wrist rigidity and tremor (using the corresponding items of the motor UPDRS). Subjects rested in a chair with their eyes open. They were asked to remain quiet and still, and rest was confirmed by visual inspection. Periods of voluntary movement detected by the examiner on visual inspection were noted and excluded from further analysis. However, periods of rest tremor or dyskinesias were not rejected.

In the United Kingdom bilateral STN LFPs were low pass filtered at 1 kHz, sampled with a frequency of 2,048 Hz and recorded in a monopolar configuration (contacts 0–4) through a commercial amplifier (TMSI Port 1, TMS International B.B., The Netherlands). Subsequently they were converted to 3 bipolar montages by subtraction of the signals from adjacent contacts and high-pass, notch and low-pass filtered at 1 Hz, 49–51 Hz and 100 Hz, respectively.

In Germany LFPs were recorded directly from bipolar pairs of adjacent electrode contacts bilaterally. Data were amplified and filtered at 1–250 Hz using a custom-made, high impedance amplifier (which had as its front end input stage the INA128 instrumentation amplifier, Texas Instruments Incorporated 12500 TI Boulevard Dallas Texas, USA) and recorded through a 1401 A–D converter (Cambridge Electronic Design) onto a computer using Spike2 software (Cambridge Electronic Design). Signals were sampled at ≥625 Hz. Given the differences in recording techniques we only contrasted normalised measures (percentage total power, coherence and correlation) and not absolute power levels in later analysis.

The first 74 s (minimum recording length across subjects) was taken from each recording, separately off and on levodopa, to allow for comparison of coherence within and between subjects.

### Data Analysis and Statistics

Bipolar LFP time series were imported into Matlab (version 7.10) and analysed with custom written scripts evoking functions from the Matlab signal processing toolbox. Power spectral analysis was calculated using an average periodogram method with a 1 s Hamming window and 50% overlap yielding a frequency resolution of 1 Hz. Low frequencies (<4 Hz) were ignored due to possible contamination by movement artifacts such as those due to dyskinesias. Power was normalized as the percentage total power between 5 and 90 Hz (excluding a 49–51 Hz band contaminated by European line noise) prior to visualization and further analysis. The power spectra of all six bilateral bipolar channels were analysed for peaks in the beta range (13–32 Hz, function - ‘findpeaks.mat’ - returns local maxima, defined as a bin which is larger than its two neighbours, with a set minimum inter peak distance of 4 Hz). For the power spectral analysis and beta peak count, the presence and position of the beta peak was then confirmed by visual inspection of the power spectral density function. The bipolar channel on each side with the highest amplitude beta peak was used for further analysis, in line with evidence that beta activity is focused in the STN, especially in its dorsolateral ‘motor’ portion [23], [24]. The beta band was subdivided into low (13–20 Hz; beta 1) and high (21–32 Hz; beta 2) ranges for further analysis [12], [13], [25]. Co-modulation of STN activities across the hemispheres in the gamma band is the subject of another study.

### Coherence

The squared coherence of the bilateral signal pairs was determined using a mean squared average periodogram method (1 s Hanning window, 0% overlap) which again yielded a 1 Hz frequency resolution. The theoretical upper limit of the 95% confidence interval (CI) for the coherence was defined as 1−p^1/(N−1)^ where p = 0.05 and N was equal to the number of windows in the periodogram [26]. By itself, this method does not compensate for multiple comparisons across different frequencies. Accordingly, we defined a significant elevation/peak as one that exceeded the 95% confidence limit over at least two consecutive frequency bins. As our frequency range of interest was the beta band (20 points, 13–32 Hz), this restricted the family wise rate to 0.05 (Type 1 error p = probability of obtaining two adjacent points in the beta band greater than 95% CIs by chance = (0.05^∧^2)*20 = 0.05). We also confirmed that this approach was conservative by demonstrating that the 95% CI derived above exceeded those derived from a surrogate dataset using a previously described method [10]. For this second method the phase components of the two signals were randomly phase shuffled in the frequency domain before using the inverse Fourier transform to recreate 1000 surrogate pairs of time series that were then analysed to determine the empirical sampling distribution of the coherence. Statistical significance was assessed in each frequency bin by comparing the actual coherence with the 100(1−p) percentile from the sampling distribution, where p = 0.05.

### Amplitude Co-modulation

**Figure 1:**
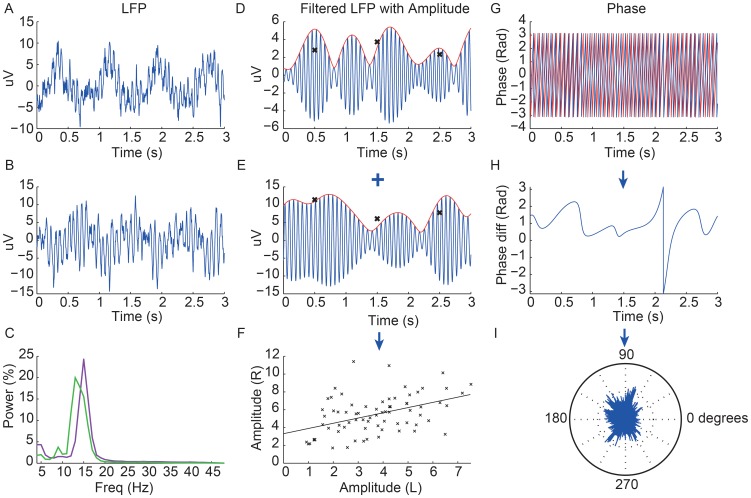
Data from one subject off levodopa showing LFPs with extracted amplitude and phase. The first column shows 3(A = left STN, B = right STN) and their respective power spectra (C; left STN - purple, right STN - green). The second column shows the LFP pass-band filtered around the corresponding beta peak (blue) of each STN (D = left STN, E = right STN) with the amplitude shown in red. The crosses show the average amplitude for each 1 second window and the final graph shows the correlation of these 1 s average amplitudes across the two sides over 74 s duration record, with a linear regression line through them (F). The r value of this linear regression line is taken as the value of the amplitude co-modulation for any given subject. In this example r = 0.57, p<0.001. The right column shows the superimposed phase of the two LFP signals (red = left STN and blue = right STN) over 3 s (G) with the phase difference over this period shown below (H). A rose plot underneath shows the proportion of phase difference vectors at all points for the whole recording around the unit circle (I). The length of the average of these vectors is then taken as the value of the phase locking value (PLV), which in this case was 0.22. Note low frequency oscillations at about 1 Hz likely to be cardiac pulse artefact in A and B. Despite this, modulations in the amplitude envelopes of the beta band filtered LFP activity shown in D and E are not time-locked to the low frequency cardiac pulse artifacts in 1A and B.

The amplitude envelope of the signal was assessed for all frequencies by taking the modulus of the pass-band filtered and Hilbert transformed signal. Pass-band filtering was performed in both the forward and reverse directions, to achieve zero phase distortion, using a fourth order Butterworth filter with a pass-band of 1 Hz. Co-modulation of amplitude was determined by dividing the amplitude envelope into windows and averaging the amplitude within each window. The co-modulation index was defined as the Pearson’s correlation coefficient of the averaged windowed amplitude series from the two STNs (Fig. 1). This was repeated for all frequencies within the beta range. We tested windows of 0.5, 1, 5 and 15 s duration and found no significant difference between correlations. Only data for 1 s windows are presented. We determined whether the co-modulation index was different to that expected by chance by comparison with a surrogate dataset. The sampling distribution for the amplitude co-modulation was determined by calculating the correlation coefficient at each frequency between the windowed averaged amplitudes for 1000 pairs of time shuffled pairs of time series. Statistical significance was assessed in each frequency bin by comparing amplitude co-modulation with the sampling distribution of surrogate data, as above.

### Phase Synchronization

Phase information was extracted following passband filtering and Hilbert transformation of both time series at all frequencies (Fig. 1). Phase synchronization was calculated according to the phase locking value (PLV) method, also known as the mean phase coherence or synchrony factor, which is a measure of how the relative phase is distributed over the unit circle [27], [28]. The phase difference of the two unwrapped phase signals was computed and the modulus (2 π) taken to create a time series of phase differences at all points in time on the unit circle. The phase difference vectors at all points in time were then averaged and the length of the average vector (between 0 and 1) was taken as the measure of phase synchronization. This was repeated for all frequency pairs:
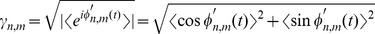
where φ_n,m_(t) represent the relative phase difference at all points in time, *t* for the two signals n and m. The statistical significance of this phase locking value (PLV) was again estimated using a surrogate dataset. The sampling distribution of the PLV was determined by randomly shuffling the time points of the two signals and calculating the PLV between these two time-shuffled series. This was repeated 1000 times and the 95% CI for the PLV determined as above. Note that frequency co-modulation across the two STN was not studied here, as it does not directly relate to coherence.

Statistical analyses were performed using SPSS version 19 (SPSS Inc., Chicago, IL, USA) and Matlab (version7.10) statistics toolbox. Data were normally distributed or transformed so that they became normally distributed (see results for relevant transforms; single sample Kolmogorov-Smirnov tests p>0.05, following False Discovery Rate (FDR) correction for multiple comparisons) [29]. Means, standard error of the means and parametric statistical analyses (including Pearson’s correlation) are presented. Repeated measures two factor ANOVAs were tested and post-hoc tests presented as significant if they survived FDR correction for multiple comparisons.

## Results

### Power Spectra

**Figure 2:**
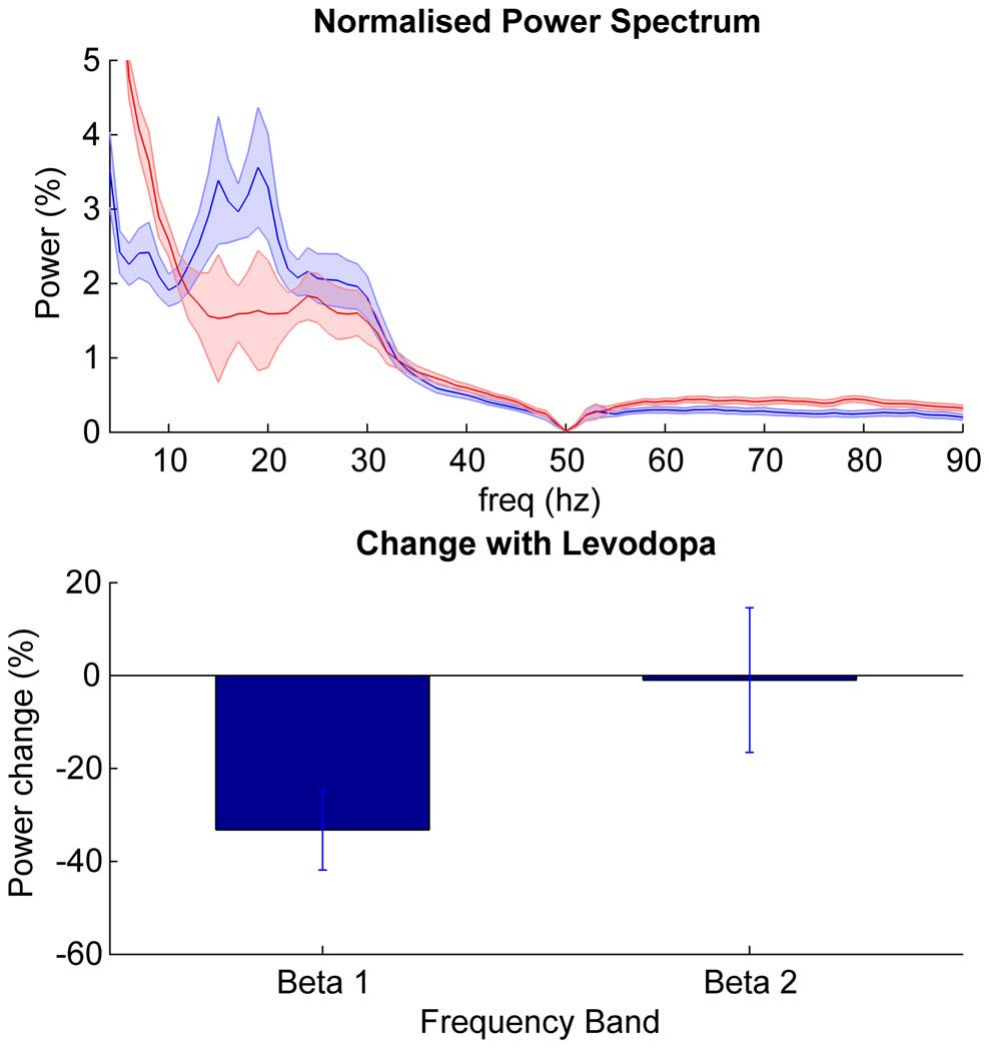
Power changes in STN. Top panel shows mean ± SEM power spectral density of all 23 subjects in the off (blue) and on (red) medication state. Bottom panel shows the mean ± SEM % change between the two states (on – off medication) in the beta sub-bands. Only the power suppression in the beta 1 band following levodopa was significant (t_22_ = −4.4, p<0.001).

Group data for patients on and off medication are presented in Fig. 2. Power spectra demonstrated beta peaks off medication on both sides in 22 subjects and on one side only in one subject. The mean beta peak frequency was 22±0.84 standard of the mean (SEM) Hz. Log transformed power was analysed by repeated measures two factor ANOVA (factors beta sub-band and levodopa) and demonstrated a main effect of levodopa (F_(df 1,22)_ = 17.5, p<0.001), but no effect of frequency band (F_(df 1, 22)_ = 2.3, p = 0.14). There was a significant interaction between frequency and levodopa (F_(df 1,22)_ = 4.6, p = 0.044). Post hoc analysis (two tailed paired t tests) revealed a reduction in beta 1 power after levodopa (t_22_ = −4.4, p<0.001) but no significant change in beta 2 (t_22_ = −1.5, p = 0.4). The beta peak frequencies across the two sides were strongly correlated (r = 0.70, p<0.001).

### Coherence

**Figure 3:**
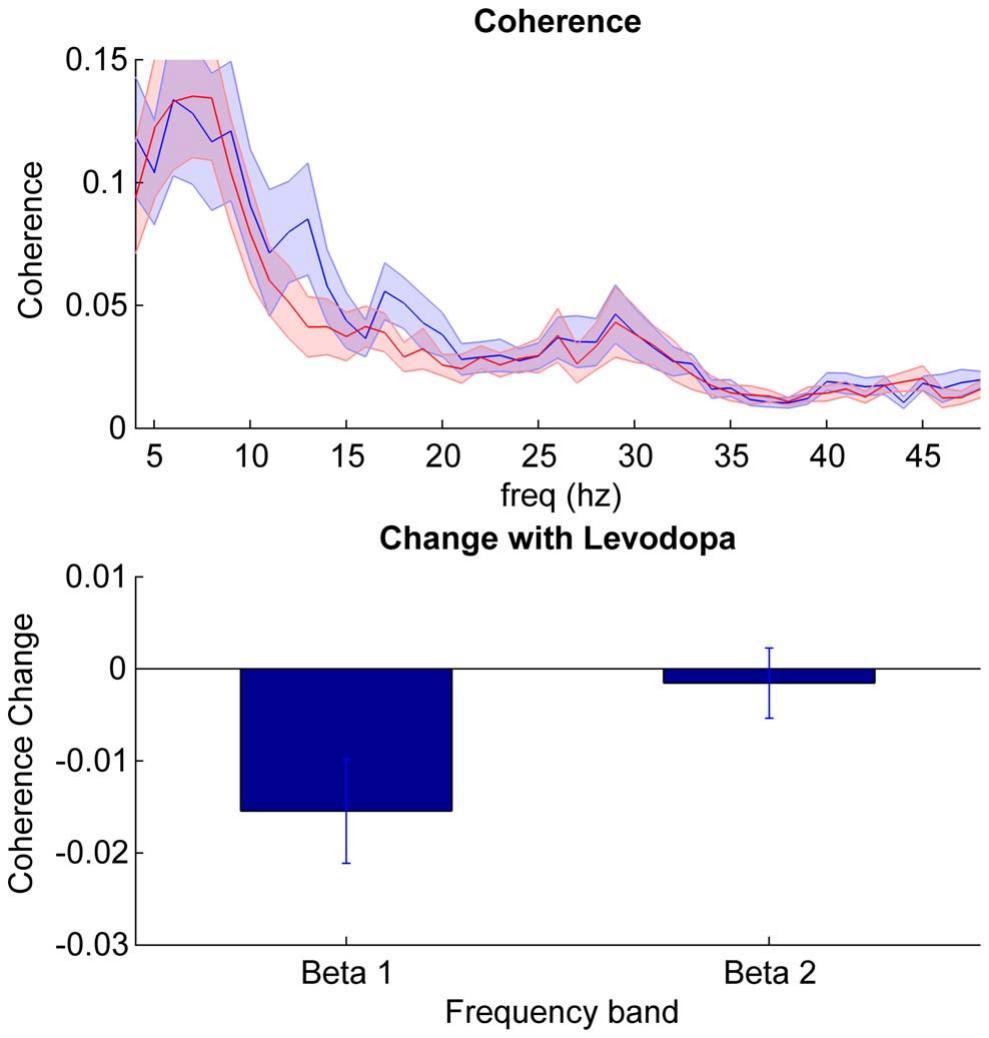
Coherence between STNs. Top panel shows mean ± SEM coherence of all 23 subjects in the off (blue) and on (red) medication state. Bottom panel shows the mean ± SEM % change between the two states (on –off medication) in the beta sub-bands. Only the coherence suppression in the beta 1 band following levodopa was significant (t22 = −2.7; p = 0.01).

Group data for patients on and off medication are presented in Fig. 3. There was significant coherence between the STN sides in the beta range in 19 out of the 23 subjects in the off medication state. The mean frequency of the coherence peak was 20.6±1.3 Hz, with a mean maximum coherence of 0.15±0.02 across the whole beta band. On levodopa the number of subjects with significant bilateral coherence dropped to 15 (mean frequency of coherence peak 23.3±1.2 Hz and mean maximum coherence 0.11±0.02 across the whole beta band). Differences in Fisher transformed coherence averaged across beta sub-bands were analysed with repeated measures two factor ANOVA (factors beta sub-band and levodopa) and demonstrated a main effect of levodopa (F_(df 1,22)_ = 4.5, p<0.046) but no significant effect of frequency (F_(df 1,22)_ = 3.0, p = 0.10). There was an interaction between levodopa state and frequency (F_(df 1,22)_ = 5.7, p = 0.026). Post hoc tests confirmed a significant reduction in beta 1 coherence with levodopa (t_22_ = −2.7; p = 0.01), but no change in beta 2 coherence (t_22_ = −0.4; p = 0.69).

### Amplitude Co-modulation

**Figure 4:**
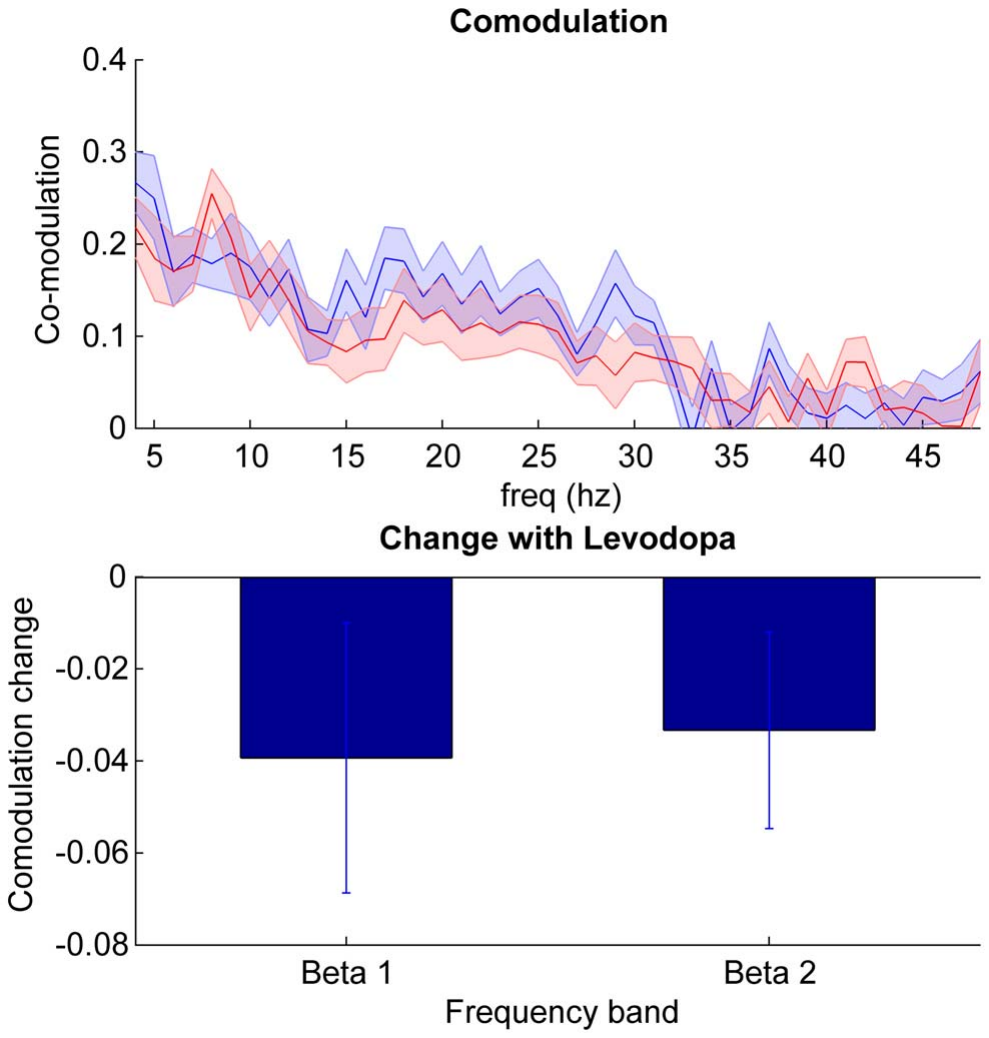
Amplitude co-modulation between STNs. Top panel shows mean ± SEM amplitude co-modulation of all 23 subjects in the off (blue) and on (red) medication state. Bottom panel shows the mean ± SEM % change between the two states (on – off medication) in the beta sub-bands. There was no significant effect of levodopa, frequency band or interaction between the two (see results).

Group data for patients on and off medication are presented in Fig. 4. The correlation between the fluctuations in the amplitude envelopes in the beta range on the two sides was calculated independent of phase and compared against a surrogate dataset using the same window (1 s) as for the coherence analysis. We found that off medication 17 subjects showed a significant positive beta amplitude co-modulation peak with an average r value of 0.40±0.03 and mean peak frequency of 21.7±1.3 Hz across the whole beta band. This reduced to 10 subjects on medication with an average peak r of 0.34±0.02 and mean peak frequency of 22.1±1.2 Hz. Group analysis of Fisher transformed correlation coefficients with repeated measures ANOVA (factors beta sub-band and levodopa) did not demonstrate a main effect of either levodopa (F_(df 1,22)_ = 3.0, p = 0.10) or frequency (F_(df 1,22)_ = 1.5, p = 0.23), nor any interaction between levodopa and frequency (F_(df 1,22)_ = 0.04, p = 0.84).

### Phase Locking Value

**Figure 5:**
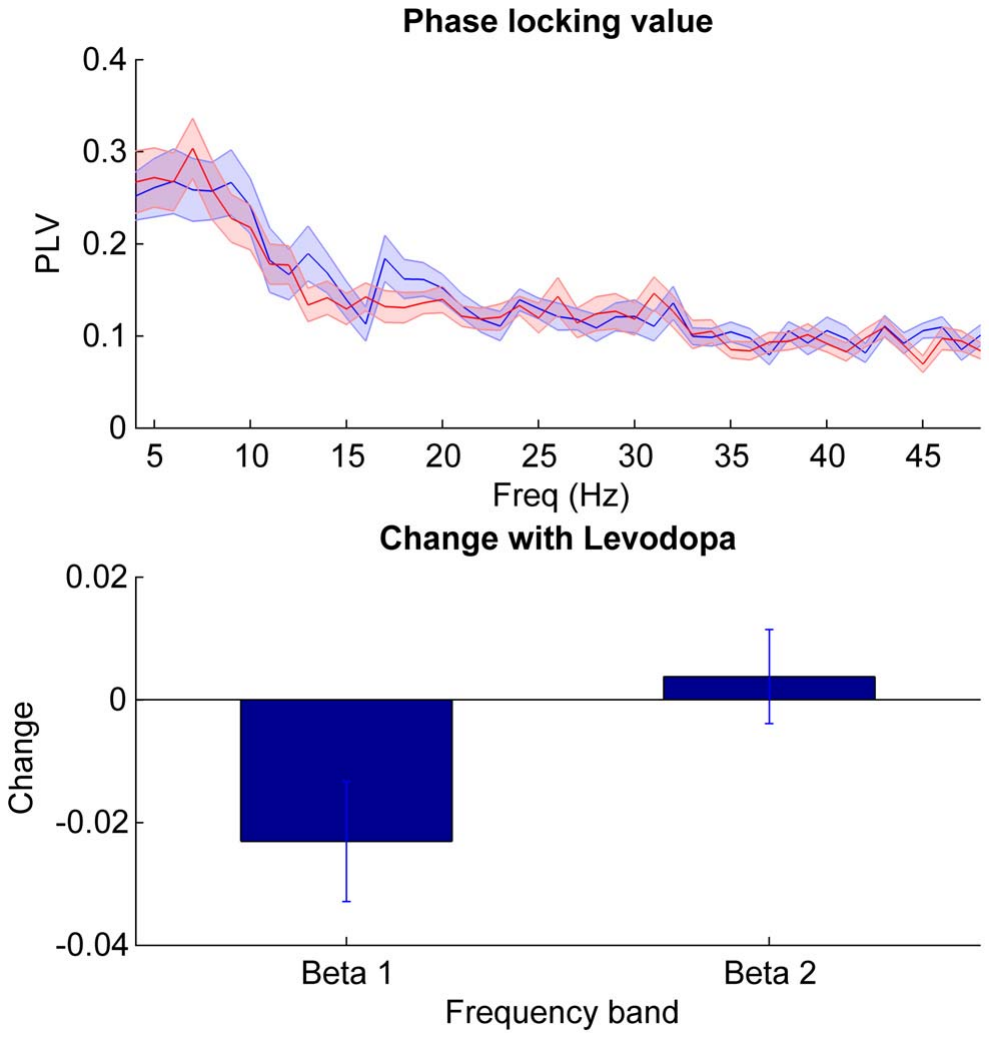
Phase synchronisation between STNs. Top panel shows mean ± SEM amplitude PLV of all 23 subjects in the off (blue) and on (red) medication state. Bottom panel shows the mean ± SEM % change in PLV between the two states (on – off medication) in the beta sub-bands. Only the beta 1 band PLV was suppressed following levodopa (t_22_ = −2.8, p = 0.01).

**Figure 6:**
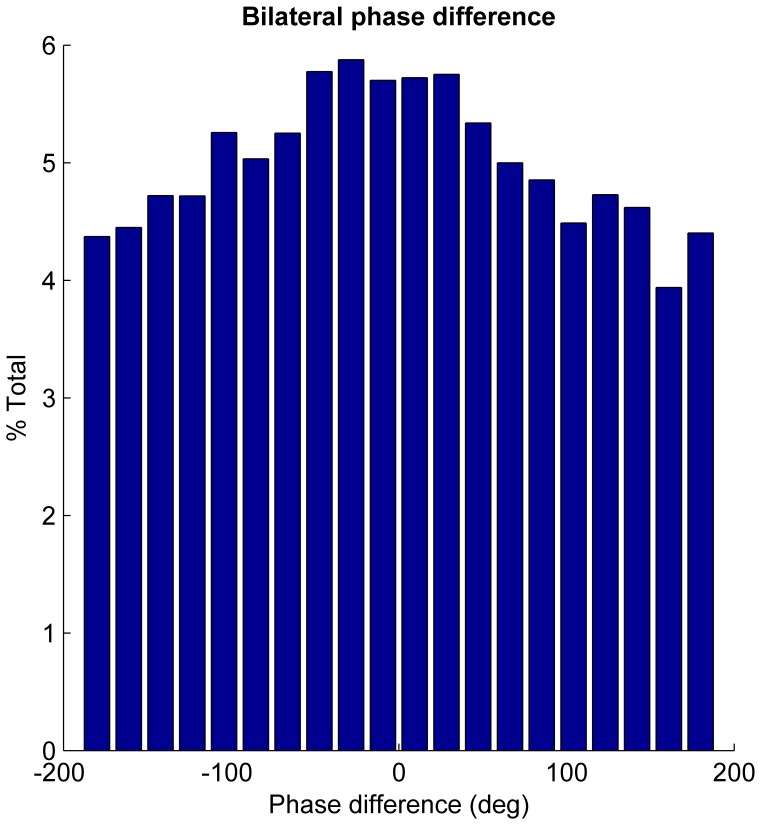
Histogram of beta phase differences between bilateral STN. Histogram of all phase differences across 23 subjects at peak beta frequency off medication, demonstrating predominance of zero phase lag.

Pure phase synchronization, independent of amplitude co-modulation, was assessed using the phase locking value (PLV) in the beta range (Fig. 5). This revealed significant phase locking in 16 subjects off medication, with a mean peak PLV of 0.29±0.02 and mean peak frequency of 21.4±1.4 Hz across the whole beta band off medication. This reduced to 12 subjects and to a PLV of 0.26±0.15 following levodopa (mean peak frequency of 24.9±1.4 Hz). A repeated measures ANOVA of PLV (factors beta sub-band and levodopa) revealed a borderline main effect of frequency (F_(df 1,22)_ = 4.3, p = 0.051) and an interaction between levodopa and frequency (F_(df 1,22)_ = 7.3, p = 0.01). There was no main effect of levodopa (F_(df 1,22)_ = 1.8, p = 0.20). Post hoc testing demonstrated a reduction of phase locking with medication in the beta 1 range (t_22_ = −2.8, p = 0.01) but no effect in beta 2 (t_22_ = 0.8, p = 0.41). Rayleigh’s test confirmed a non-uniform distribution of phase differences between STN beta oscillations around the unit circle (p<0.001) and a histogram of phase differences demonstrated a peak centred around zero phase lag (Fig. 6). The mean phase lag (−0.026 rad/−14°) was found to be not significantly different from zero (one sample circular t-test, n = 23, p = 0.21).

## Discussion

In this study we investigated the bilateral functional connectivity of the subthalamic nuclei on and off levodopa in a cohort of patients with PD. We confirmed the previously described subthalamic beta range coherence between the left and right STNs in the off medication state [10], and further demonstrated an acute and relatively selective suppression of phase locking in the lower beta range in response to levodopa. This suppression mirrored the effect of levodopa on local power in the STN in this sub-band. Although amplitude co-modulation of beta was present between the two sides, this was not significantly affected by levodopa in either the lower or upper beta range.

A distributed cortical beta network has been shown previously to be present in Parkinson’s disease, to be modulated by dopamine and to correlate with disease severity [11]. This study is the first to examine the beta network subcortically in response to dopamine. Our data parallel that found at the cortex and therefore extends the concept of distributed, dopamine responsive, beta synchronisation to subcortical structures. Widespread beta synchronisation could represent a purely pathological response to dopamine withdrawal. However, it should be noted that despite a robust reduction in beta 1 coherence at the group level in response to levodopa, there still remained statistically significant residual beta coherence peaks in the majority of subjects. Although this could be explained by an insufficient levodopa dosage in some patients it also raises the possibility that widespread beta connectivity is not simply pathological but plays a physiological role, at least at lower levels of coherence.

From coherence alone it is difficult to deduce the exact physiological mechanism of connectivity and, specifically, whether coupling takes the form of amplitude co-modulation and/or phase synchronization. Both have been suggested as providing potential coding schemes [30]. Phase synchronization, in particular, leads to coordinated neuronal firing and the synchronous arrival of multiple excitatory post-synaptic potentials (EPSPs) onto target neurons. Given the short half-life of EPSPs this temporal summation promotes efficient information transfer from one population to the next [31], [32]. However, when exaggerated as in PD, phase synchronisation potentially leads to a loss of information coding space or entropy [33]. Although it remains to be proven whether amplitude co-modulation is exaggerated in PD, this form of interaction may also have physiological bounds, and when pronounced across mesoscopic signals like the LFP may necessarily entail attenuation of time varying amplitude modulation on finer spatial scales. Here, we provide evidence that interactions in the beta band occur within a distributed bilateral network, as well as at the local basal ganglia level [34]. Only that cross-hemispheric interaction expressed through phase synchronization (and, secondarily to this, through coherence) was significantly attenuated by dopaminergic input, and even then, only in the low beta range. Amplitude co-modulation in the same frequency band was relatively unaffected by acute dopaminergic therapy. Dopamine may therefore primarily modulate functional connectivity between the STN on the two sides through changes in the co-ordination of phase between sites. This ability of dopamine to acutely change functional connectivity with a time-course that excludes structural change has previously been noted in ipsilateral basal ganglia-cortical circuits, both empirically and in modeling studies [35]–[37].

This study is consistent with a growing body of evidence that demonstrates a functional dissociation between low and high beta range activities, highlighted by their differential sensitivity to dopaminergic therapy [12], [13] and the preferential coherence between the STN and cortex in the upper beta range [3], [25]. Here we extend those studies which examined local synchrony as found in the single site LFP recordings and show that distributed bilateral coherence is characterized by a fixed, dopamine unresponsive peak in the high beta range and a second dopamine reactive frequency band in the low beta range.

The anatomical pathways sub-serving the functional connectivity between the STNs are currently unclear as there is no known direct anatomical interhemispheric connection between these nuclei in the primate [38], [39]. Although limited activity in the contralateral STN can be evoked at very short-latency by stimulation of the ipsilateral nucleus, possibly through stimulation of fibres of passage [40], [41], the bulk of modulatory effects of stimulation of one STN upon the other are of much longer latency than the temporal difference between STNs reported here, and are compatible with indirect communication between the two STNs [40]. Potentially, the bilateral coherence shown here and elsewhere [10] could also be mediated by indirect poly-synaptic pathways, although the centering of phase lags between the STN near zero places an upper limit on the delays involved in such indirect connectivity. Alternatively the two STN could be synchronized through common input to both from one or more additional structures. One candidate common input might be cerebral cortical activity in the beta frequency band, given that this is coherent with and leads that in STN and so may plausibly drive subcortical oscillations in the beta range [2], [3], [42]. Contralateral cortico-striatal connections have been identified and these, through the indirect pathway, could provide the anatomical underpinning for this common beta input [43], [44]. An alternative might be bilateral projections in the hyperdirect pathway, although these are as yet unconfirmed in the human. However, as previously noted, coherence between the cortex and STN preferentially occurs in the upper beta range [3], [25], so that a common input from cortex may not necessarily explain the coupling between the two STN at lower beta frequencies.

With the advent of aDBS, the question of whether basal ganglia on the two sides act as a separate or a single functional network is important from a translational perspective. Given recent work showing efficacious aDBS triggered off beta amplitude in PD patients [17] it is necessary to know whether any coupling of amplitude across the two sides is sufficient to control bilateral aDBS from ipsilateral recordings. We find that, although statistically significant, the peak level of co–modulation was low and therefore unilateral monitoring would not be appropriate for bilateral triggering of stimulation off individual beta bursts [17].

## Conclusion

This study is the first to demonstrate the effect of levodopa on subcortical beta functional connectivity in PD with levodopa suppressing coherence in the low beta band. We show that this beta connectivity is driven by both significant amplitude co-modulation and phase synchronisation but that it is the phase effects that are predominantly modulated by dopamine. Bilateral coherence is also characterized by a functional dissociation of the high and low beta sub-bands in response to levodopa with a fixed unreactive peak in the high beta range and a dopamine responsive frequency region in the low beta range. Coupling, although robustly significant, was in absolute terms relatively weak and thus with respect to progressing aDBS from unilateral to bilateral stimulation, the two basal ganglia networks may have to be approached separately with independent sensing and stimulation.
